# Effects of Anionic Groups on Structural and Luminescent Properties of ZnO:Sm^3+^ Phosphors Synthesized via Combustion Method

**DOI:** 10.3390/molecules31020206

**Published:** 2026-01-07

**Authors:** Edwin Tumelo Maleho, Machaba Leanyatsa Abraham Letswalo, Buyisiwe M. Sondezi

**Affiliations:** Department of Physics, University of Johannesburg, Johannesburg ZA-2006, South Africa; tumelomaleho7@gmail.com (E.T.M.); bmsondezi@uj.ac.za (B.M.S.)

**Keywords:** phosphors, anionic group, Sm^3+^ ions, luminescence, combustion synthesis

## Abstract

BO_3_^3−^, PO_4_^3−^, and SO_4_^2−^ anionic groups were used to study their effects on the structure and luminescence of Sm^3+^-doped ZnO. ZnO, ZnO:Sm^3+^, ZnO, Zn_4_B_6_O_13_:Sm^3+^, and Zn_2_P_2_O_7_:Sm^3+^ phosphors were successfully synthesized via combustion synthesis. While BO_3_^3−^ and PO_4_^3−^ ions led to the formation of new crystalline phases, the sulfate precursor decomposed during synthesis, yielding ZnO with only minor surface sulfur traces. The XRD results revealed the formation of wurtzite crystal structures in the ZnO, ZnO:Sm^3+^, and ZnO-SO_4_:Sm^3+^ samples, while a complete change of structure was observed after the incorporation of borate (BO_3_^3−^) and phosphate (PO_4_^3−^) ions into ZnO:Sm^3+^ to Zn_4_B_6_O_13_:Sm^3+^ and Zn_2_P_2_O_7_:Sm^3+^, respectively. The structures for borate and phosphate ions were confirmed as cubic (Zn_4_B_6_O_13_) and monoclinic (Zn_2_P_2_O_7_) crystal structures, respectively. The morphological studies of ZnO:Sm^3+^ and ZnO-SO_4_:Sm^3+^ were characterized by aggregated particles with different shapes and sizes. Zn_4_B_6_O_13_ and Zn_2_P_2_O_7_ samples were characterized by having cubic and rough surfaces, respectively. The oxidation state of the Sm ions was confirmed by XPS analysis. The photoluminescence studies revealed a broad-band emission for the ZnO:Sm^3+^ and ZnO-SO_4_:Sm^3+^ materials and characteristic Sm^3+^ emissions (from the ^4^G_5/2_ level to lower states ^6^H_J_ (J = 5/2, 7/2, 9/2, and 11/2)) for the Zn_4_B_6_O_13_ and Zn_2_P_2_O_7_ samples. Enhanced emissions were observed after the incorporation of anionic group systems. The most intense PL emission was observed from the Zn_4_B_6_O_13_ phosphor material. The CIE calculations revealed that the best color purity results were from Zn_4_B_6_O_13_, which lay in the orange region with 98% color purity.

## 1. Introduction

Solid-state lighting in the form of LEDs has been favored in comparison to incandescent and fluorescent lamps, and this is because of interesting characteristics, such as a higher brightness, low power consumption, high efficiency, and being environmentally friendly [1,2,3,4]. The production of light by these materials depends on selective phosphor materials emitting light in the visible region by a UV excitation source [4]. Recently, w-LEDs have been made by two methods, which include coating a blue-emitting InGaN chip with a yellow-emitting YAG:Ce^3+^ phosphor material [5] and combining near-UV LEDs with tri-color emitting phosphor materials. White light produced by such methods usually has some drawbacks due to the lack of a red ingredient, which leads to a low color rendering index (CRI) and high correlated color temperature (CCT) [5]. These drawbacks can be overcome by improving the red components in the selected host materials with relevant dopant ions excited by an NUV LED chip [6,7,8].

ZnO is a semiconducting material and a widely studied phosphor due to its wide utility in solar cells [9], gas sensors [10], LEDs [11], and applications in optoelectronics [12]. It has a wide direct band gap of 3.37 eV and a large binding energy of 60 meV. These characteristics make it an ideal material for incorporating dopants into the ZnO matrix. Rare earth dopants, which have partially filled 4f electrons, can be easily incorporated into ZnO to tune its optical properties. These rare earths, when incorporated into ZnO, allow parity-forbidden 4f-4f transitions and defect emissions to take place [13]. Sm^3+^(${4f}^{5}$) is a rare earth ion that is widely known as a dopant due to its strong orange-red luminescence in the visible region and has applications in high-density optical storage systems, undersea communication systems, solid-state lasers, etc. [14].

The generation of white light by combining a blue LED with a yellow phosphor, such as Y_3_Al_5_O_12_:Ce^3+^, suffers from a low color rendering index and high correlated color temperature. Attempts to solve this involve exciting the RGB phosphor by a near-UV semiconductor chip for generating white light. However, this approach is not widely used because of the ineffective absorption of n-UV by red phosphors such as Y_2_O_2_S:Eu^3+^ from the semiconductor chip [15]. Therefore, there is a need to develop a red phosphor that has effective n-UV absorption abilities with enhanced emission intensities.

Amongst other red-emitting phosphors, Sm^3+^ serves as a better choice due to its low cost [16]. In the Sm^3+^ configuration, the 4f electrons are shielded by the outer 5s and 5p orbitals, which retain the atomic character of Sm^3+^. Thus, its characteristic transitions are not affected much by the host material. This results in a strong emission from Sm^3+^ ions due to transitions such as ^4^G_5/2_ → ^6^H_J_ (J = 5/2, 7/2, 9/2, and 11/2) of the ${4f}^{5}$ energy level [17,18]. Most Sm^3+^-doped phosphors tend to show effective absorption characteristics in the wavelength 380–420 nm [19,20]. Gupta et al. [21] investigated perovskite-type YAlO_3_:xSm^3+^ with varying concentrations of Sm^3+^ for application in n-UV-energized WLEDs. Kumari et al. [22] co-doped Sm^3+^ and Eu^3+^ ions in Sr_9_Y_2_W_4_O_24_ red-emitting phosphors for effective energy transfer from Sm^3+^ to Eu^3+^. Their findings revealed that n-UV light may effectively excite the Sr_9_Y_2_W_4_O_24_:Sm^3+^/Eu^3+^ co-doped phosphor material. In recent years, it has been demonstrated by different research groups that partial replacement of anionic groups in ZnO can greatly increase light emission from the dopant ions. B. Avula et al.’s [23] and M.L.A Letswalo et al.’s [24] research works demonstrated considerable improvement in red photoluminescence (PL) from Eu^3+^ in host lattices such as calcium molybdate, double molybdate, and calcium titanium oxide. The substitution of anionic groups like SO_4_^2−^, PO_4_^3−^, SiO_3_^2−^, and BO_3_^3−^ has produced striking improvements in red PL. The mechanism of such improvement is, however, not yet clearly understood. In order to fill this gap, a study was carried out to examine the manner in which the substitution of these anionic groups increases PL intensity. Furthermore, earlier studies [23,24] discovered that partial replacement of these anions can affect the color purity of the emitted red light in phosphor materials.

In this study, ZnO, ZnO:Sm^3+^, Zn_4_B_6_O_13_:Sm^3+^, Zn_2_P_2_O_7_:Sm^3+^, ZnO-SO_4_, and ZnO-SO_4_:Sm^3+^ phosphors were synthesized via urea-assisted combustion. ZnO-SO_4_:Sm^3+^ retained the wurtzite structure of ZnO when synthesized in the presence of ammonium sulfate, which acts as a combustion modifier rather than a dopant. After annealing at 1000 °C, the products remained as ZnO, as confirmed by XRD analysis. The influence of the anions as a tuning parameter was investigated for their luminescence, optical, and structural properties to see which phosphor material produced the best luminescence of all anionic group-doped materials. This work was inspired by the work of Balakrishna and co-workers, who substituted different anions into the CaMoO_4_ host material, which, when doped with Sm^3+^ ion, produced a 30 times more intense red emission when excited by an NUV LED chip [23]. Furthermore, doping with BO_3_^3−^ and PO_4_^2−^ ions by combustion synthesis may influence the crystal structure formation of the material, and different types of borate and phosphate structures may be formed, respectively. In the case of BO_3_^3−^, some of the possible borate structures that may form include ZnB_4_O_7_, ZnB_2_O_4_, Zn_4_B_6_O_13_, or Zn_3_(BO_3_)_2_ [25,26]. Similar behavior of structural change when introducing the borate (BO_3_^3−^) unit into a different host material was observed by Tamboli et al. [27]. In their work, the crystal structure was changed from the monoclinic phase of Ba_2_Mg(PO_4_)_2_ to the rhombohedral phase of Ba_2_Mg(BO_3_)_2_ when they introduced the BO_3_^3−^ ions into Ba_2_Mg(PO_4_)_2_ material. For PO_4_^2−^ doping, possible formations of phosphate may include Zn_2_P_2_O_7_, Zn_3_P_2_O_8_, or ZnP_2_O_6_ [28,29,30]. In this work, a notable phase change was observed when introducing BO_3_^3−^ and PO_4_^2−^ ions, significantly influencing the material’s structural and optical properties. In contrast, the ZnO initially doped with SO_4_^2−^ anion maintains the same phase as the host, consistent with the findings reported by Letswalo et al. [24] using the solid-state reaction method. The structural variation induced by the introduction of BO_3_^3−^ and PO_4_^2−^ anions are analyzed by XRD and Rietveld refinement.

## 2. Results and Discussion

### 2.1. X-Ray Diffraction—Phase Identification

**Figure 1:**
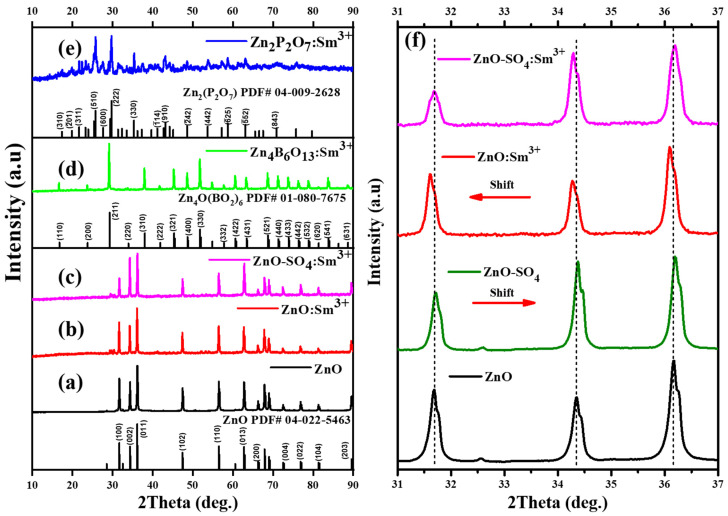
XRD patterns of (**a**) ZnO, (**b**) ZnO:Sm^3+^, (**c**) ZnO-SO_4_:Sm^3+^, (**d**) Zn_4_B_6_O_13_:Sm^3+^, and (**e**) Zn_2_P_2_O_7_:Sm^3+^ and (**f**) enlarged XRD pattern of ZnO, ZnO-SO_4_, ZnO:Sm^3+^, and ZnO-SO_4_:Sm^3+^.

**Figure 2:**
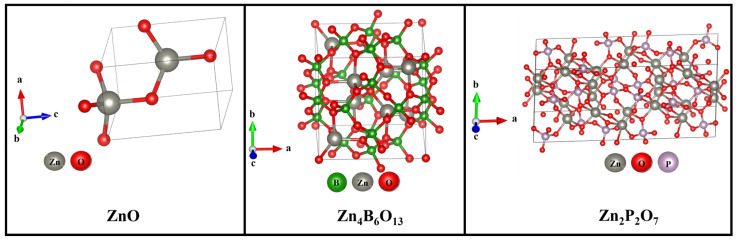
Crystal structure for ZnO, Zn_4_B_6_O_13_, and Zn_2_P_2_O_7_ materials.

Figure 1 shows the XRD patterns of ZnO, ZnO:Sm^3+^, Zn_4_B_6_O_13_:Sm^3+^, Zn_2_P_2_O_7_:Sm^3+^, and ZnO-SO_4_:Sm^3+^ phosphors synthesized via urea-assisted combustion. ZnO-SO_4_:Sm^3+^ retained the wurtzite structure of ZnO when synthesized in the presence of ammonium sulfate, which acts as a combustion modifier rather than a dopant. After annealing at 1000 °C, the products remained as ZnO, as confirmed by XRD analysis. The XRD peaks of the undoped ZnO host material (Figure 1a) correspond to those of a hexagonal crystal structure and are well matched with the standard background ZnO structure (PDF file number 04-022-5463) with a space group of P63mc [31]. These diffraction peaks were found around 2θ angles of 32°, 35°, 37°, 48°, 57°, 63°, 67°, 68°, 70°, 73°, 77°, 82°, and 87°, which are well indexed as (100), (002), (101), (102), (110), (103), (200), (112), (201), (004), (202), (104), and (203) crystal planes of the material, respectively, and whose lattice parameters are a = b = 3.25 Å and c = 5.21 Å [31]. However, when adding the other two anions (PO_4_^2−^ and BO_3_^3−^) into ZnO (Figure 1d,e), the crystal structure was altered completely from a hexagonal wurtzite crystal structure to monoclinic zinc pyrophosphate (Zn_2_P_2_O_7_) and cubic zinc metaborate (Zn_4_B_6_O_13_) crystal structures for PO_4_^2−^ and BO_3_^3−^, respectively. This behavior was not observed in the work of Balakrishna et al. [23], where they added the anions into a different host material. The diffraction peaks of the Zn_4_B_6_O_13_ phosphor material are well matched with its reference background structure (PDF file number 01-080-7675) with a space group of I-43m, with cubic lattice parameters of a = b = c = 7.48 Å [32]. These diffraction peaks were found around at 2θ angles of 17°, 26°, 30°, 35°, 38°, 42°, 46°, 50°, 55°, 58°, 62°, 65°, 70°, 72°, 75°, 77°, 80°, 82°, 85°, and 90° and are well indexed as (110), (200), (211), (220), (310), (222), (321), (400), (300), (332), (422), (431), (521), (440), (433), (442), (532), (620), (541), and (631) crystal planes of the material, respectively. The addition of the dopant Sm^3+^ into the Zn_4_B_6_O_13_ phosphor material did not change its cubic crystal structure; there were no additional peaks observed. In a similar manner, the addition of the phosphate (PO_4_^2−^) group into the ZnO matrix resulted in a change of crystal structure to a monoclinic zinc pyrophosphate crystal structure. The diffraction pattern of Zn_2_P_2_O_7_ is well matched with the background reference crystal structure (PDF file number 04-009-2628) with a space group of C2/c and with monoclinic lattice parameters of a = 6.61 Å, b = 8.29 Å, and c = 4.51 Å [28]. These diffraction peaks were found at 2θ angles of 17°, 20°, 21°, 25°, 27°, 29°, 35°, 40°, 44°, 48°, 53°, 59°, 63°, and 70°, which are well indexed as (310), ($\bar{2}$01), (311), (510), (600), (222), (330), ($\bar{1}$14), (910), ($\bar{2}$42), (442), ($\bar{6}$25), ($\bar{5}$52), and (843) crystal planes of the material, respectively. The crystal structures of ZnO, Zn_4_B_6_O_13_, and Zn_2_P_2_O_7_ materials are displayed in Figure 2.

It appears that the addition of the dopant Sm^3+^ to the Zn_2_P_2_O_7_ has no significant effect on the diffraction patterns; the monoclinic crystal structure was preserved. Figure 1f presents the magnified 2θ region (31–37°) of the ZnO-based materials. It is evident that the incorporation of ammonium sulfate, intended to introduce SO_4_^2−^ ions into the ZnO lattice, did not result in any significant structural modification and was not successful. This is likely because sulfate species decompose at temperatures below the synthesis temperature (600 °C), leading to volatilization of sulfur as gaseous oxides such as SO_3_. Consequently, ZnO remains the dominant crystalline phase. The slight and negligible peak shift observed may be attributed to minor surface adsorption or residual impurities rather than actual lattice substitution, as shown in Figure 1f [24,33]. The Sm^3+^ ion on the other side causes a 2θ shift to the left. The shift to the lower angle suggests that the Sm^3+^ ion with a larger ionic radius (1.09 Å) substitutes the smaller-sized Zn^2+^ (0.74 Å) [34]; this will cause an increase in the interplanar d-spacing [22]. In order to retain Bragg’s law 2dsinθ = nλ, where nλ remains constant, the angle θ should shift to lower angles [21].

**Figure 3:**
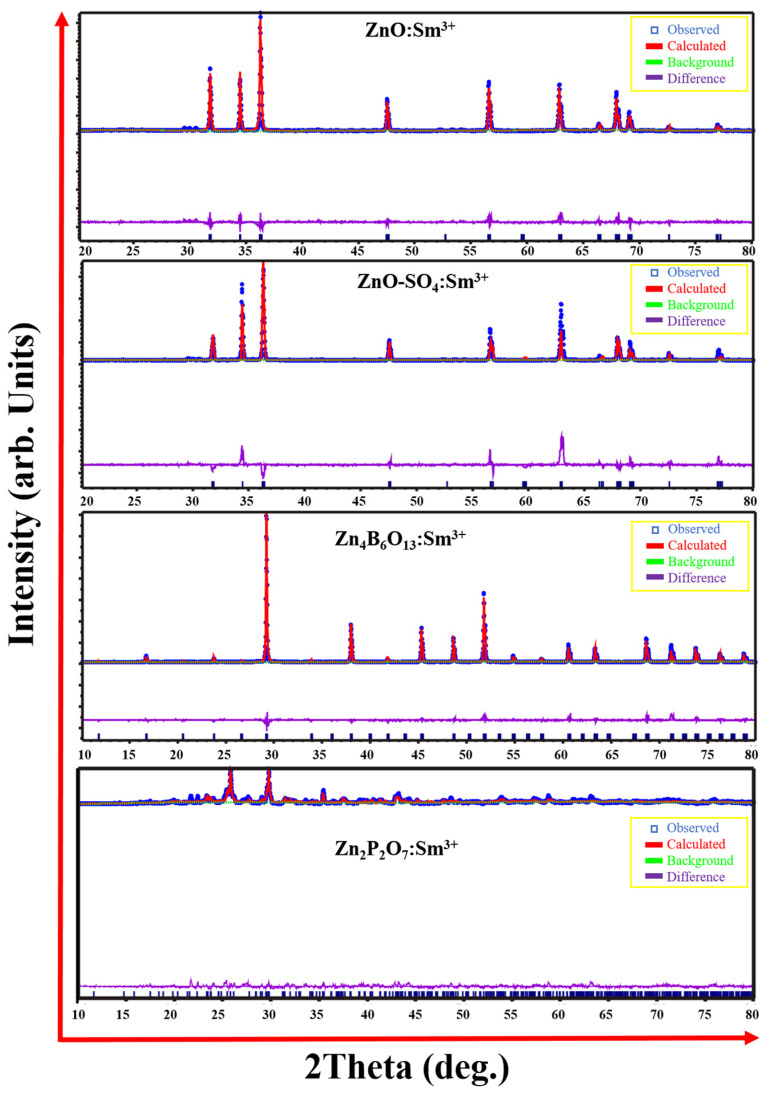
Rietveld analysis of ZnO, ZnO:Sm^3+^, ZnO-SO_4_:Sm^3+^, Zn_4_B_6_O_13_:Sm^3+^, and Zn_2_P_2_O_7_:Sm^3+^ phosphors.

The average crystallite sizes were estimated from the XRD patterns using the Debye–Scherrer equation:

(1) $$D=\frac{k\lambda }{\beta cos\theta }$$

where D is the average crystallite size and k is the shape factor, while β is the full width at half maximum (FWHM) and *θ* is the diffraction angle for the diffraction peak [35]. Table 1 below presents the estimated crystallite sizes. The materials with a ZnO hexagonal crystal structure show similar crystallite sizes; however, for the ZnO-SO_4_:Sm^3+^ sample, the crystallite size is lower by as much as 10 nm. The smaller crystallite size agrees with the XRD peak broadening of the ZnO-SO_4_:Sm^3+^ compared to other ZnO samples. All numerical values are presented with estimated uncertainties derived from the instrumental resolution and data fitting precision. The crystallite size error (±0.8–1.0 nm) was determined from multiple peak broadening fits using the Scherrer equation. Lattice parameters and cell volumes have uncertainties of ±0.001–0.005 Å and ±0.1–1.5 Å^3^, respectively. To obtain a clear indication of the changes that occur in the structure when introducing the dopant ions, the structural refinements were performed using the Rietveld refinement (RR) method in the Expo-2014 program (Figure 3). The agreement factors and the cell parameters derived from refinement are tabulated in Table 2. After the introduction of Sm^3+^ ions into the crystal structure, the ZnO-SO_4_:Sm^3+^ retained the hexagonal phase of the host material, and the cell volumes were very close. Substituting BO_3_^3−^ and PO_4_^2−^ ions completely formed new phases. This behavior was not observed when substituting with sulfate precursors, mainly because the sulfate decomposes below the synthesis temperature (600–1000 °C), resulting in the evolution of sulfur trioxide (SO_3_) gas rather than retention of SO_4_^2−^ ions in the ZnO lattice. The goodness of fit (χ^2^) plays a significant role in the process of Rietveld refinement and is described as follows [36]:

(2) $$\chi^{2}={\left(\frac{R_{wp}}{R_{ex}}\right)}^{2}$$

where R_wp_ is the weighted profile factor and R_exp_ is the expected profile factor. Based on the findings of the RR analysis, the calculated χ^2^ values for the prepared phosphors exhibit a range of values, suggesting the presence of favorable characteristics. Any minor discrepancies observed are likely attributed to internal strain, as the relevant peaks are more accurately described by a Gaussian function. The RR analysis indicates that the crystallographic characteristics, volumes, and refinement factors of the solid samples are highly comparable. The fitting graphs correspond well with the experimental outcomes, verifying the hexagonal structure of ZnO, ZnO:Sm^3+^, and ZnO-SO_4_:Sm^3+^, cubic structure of Zn_4_B_6_O_13_:Sm^3+^, and monoclinic framework of Zn_2_P_2_O_7_:Sm^3+^ phosphors, which closely align with the crystal structure information of the respective standard JCPDS cards.

### 2.2. Morphological Study

**Figure 4:**
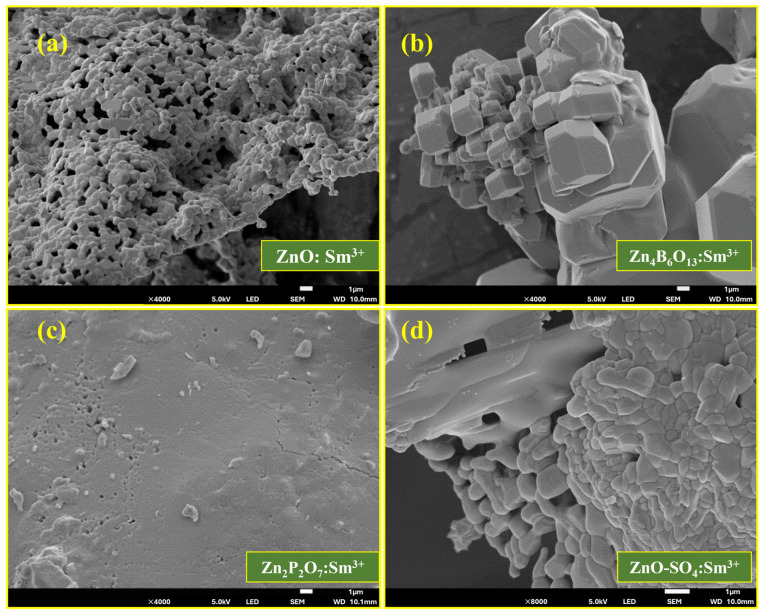
SEM images of (**a**) ZnO:Sm^3+^, (**b**) Zn_4_B_6_O_13_:Sm^3+^, (**c**) Zn_2_P_2_O_7_:Sm^3+^, and (**d**) ZnO-SO_4_:Sm^3+^.

**Figure 5:**
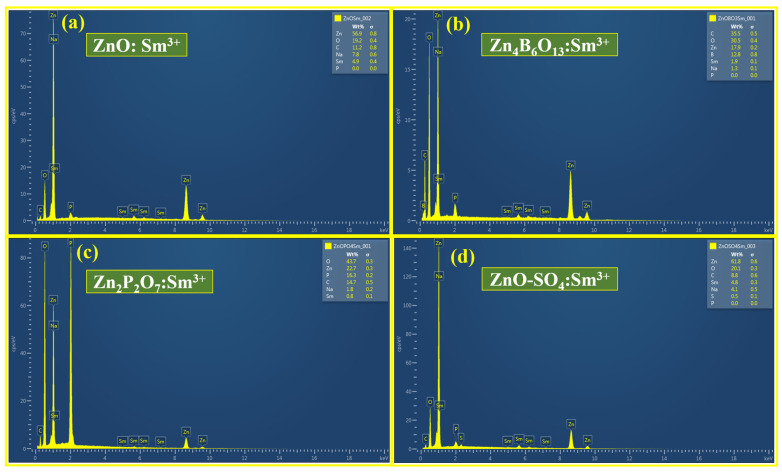
EDS spectra of (**a**) ZnO:Sm^3+^, (**b**) Zn_4_B_6_O_13_:Sm^3+^, (**c**) Zn_2_P_2_O_7_:Sm^3+^, and (**d**) ZnO-SO_4_:Sm^3+^.

**Figure 6:**
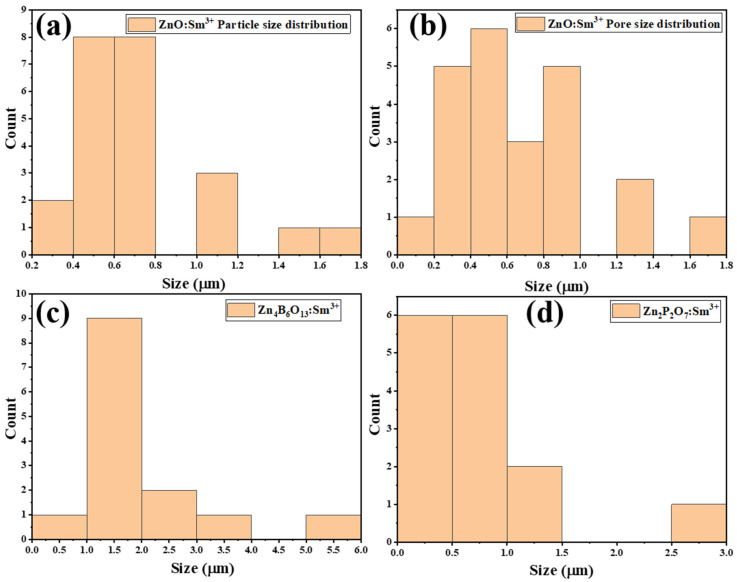
Average particle size of (**a**) ZnO:Sm^3+^, (**b**) average pore size of ZnO:Sm^3+^, and average particle size of (**c**) Zn_4_B_6_O_13_:Sm^3+^ and (**d**) Zn_2_P_2_O_7_:Sm^3+^.

Figure 4 and Figure 5 show the SEM images and EDS spectra, respectively, of the ZnO:Sm^3+^, Zn_4_B_6_O_13_:Sm^3+^, Zn_2_P_2_O_7_:Sm^3+^, and ZnO-SO_4_:Sm^3+^ samples. Figure 4a shows Sm^3+^ (1mol%)-doped ZnO phosphors with a cluster type of spherical particles with different sizes and shapes. A sponge-like morphology made up of fused micro-grains with pore sizes centered at (0.651 μm) and an average grain diameter of (0.721 μm) is revealed by the SEM image. Figure 6a,b shows the particle sizes and pore sizes distributions of the ZnO:Sm^3+^ material. A high specific surface area is suggested by the open porous network, which is consistent with quick nucleation and aggregation during synthesis [37]. Due to structural change, a completely different type of morphology was observed for the Zn_4_B_6_O_13_:Sm^3+^ material (Figure 4b). This phosphor is characterized by clustered cubic types of particles with sharp edges and smooth surfaces. Under the synthesis conditions used, SEM (Figure 4b) shows well-defined faceted microcrystals with a dominant cubic/rectangular habit and an average particle size of 1.981 μm, indicating robust crystal growth and high crystallinity. The typical size distribution of this material is presented in Figure 6c. This type of morphology is typical of Zn_4_B_6_O_13_:Sm^3+^ and is in good agreement with the XRD results. The EDS spectra of ZnO:Sm^3+^ and Zn_4_B_6_O_13_:Sm^3+^ are represented in Figure 5a,b, respectively. The morphological structures of the Zn_2_P_2_O_7_:Sm^3+^ and ZnO-SO_4_:Sm^3+^ phosphor materials are represented in Figure 4c,d, respectively. Zn_2_P_2_O_7_:Sm^3+^ materials are characterized by rough surfaces with scattered particles of irregular shapes and sizes across the surface. These materials have some pores on the surface, which may be because of escaping gases during the synthesis process. Similarly, ZnO-SO_4_:Sm^3+^ (Figure 4d) phosphor has aggregated particles throughout the surface with different shapes and sizes. The pores observed in these materials are due to escaping gases during fabrication. The microstructure of Zn_2_P_2_O_7_:Sm^3+^ (Figure 4c) is primarily smooth and compact, with sparse granular features approximately 0.767 μm on average, suggesting a denser morphology that can reduce surface-related non-radiative recombination. The sparse granular distribution of Zn_2_P_2_O_7_:Sm^3+^ is represented in Figure 6d. The EDS spectra for Zn_2_P_2_O_7_:Sm^3+^ and ZnO-SO_4_:Sm^3+^ phosphors shown in Figure 5c,d, respectively, confirm the presence of all the individual elements in the respective phosphor material. For the ZnO-SO_4_:Sm^3+^ material, the EDS spectrum reveals a very low sulfur content (≈0.5 wt%). This low S content can be attributed to the synthesis conditions. Although ammonium sulfate was introduced as a precursor to supply sulfate ions intended to act as dopants, the high processing temperature likely caused thermal decomposition of sulfate species, resulting in the release of volatile gases such as SO_2_ or SO_3_ [38,39]. The EDS spectra of all materials reveal the presence of carbon in the samples, which did not form part of the precursors. The presence of carbon is due to the carbon coating that was used during analysis, and the unexpected detection of Na in all samples may be due to surface contamination.

### 2.3. FTIR Measurements

**Figure 7:**
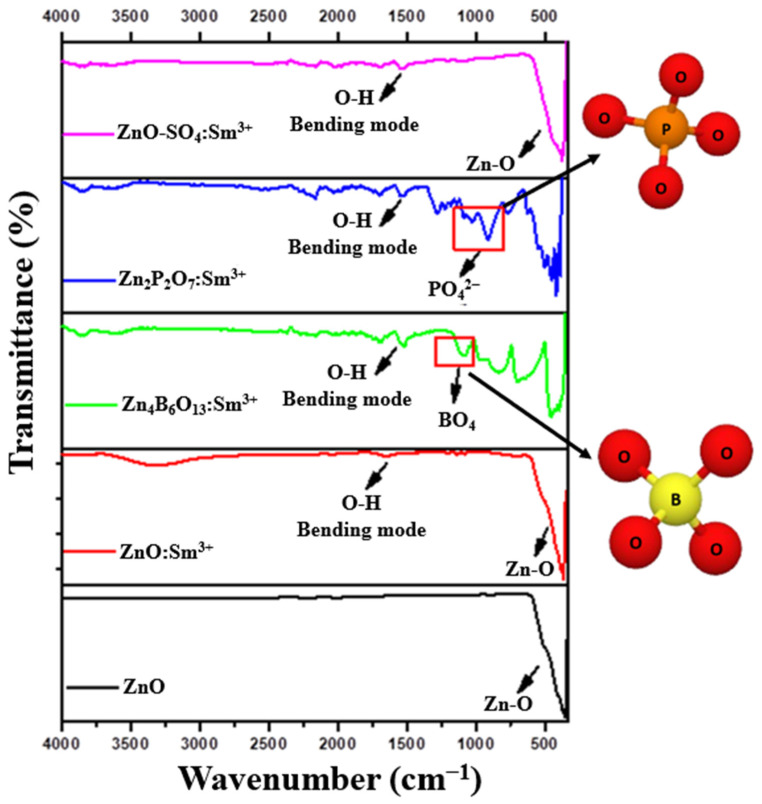
FTIR spectra of ZnO, ZnO:Sm^3+^, Zn_4_B_6_O_13_:Sm^3+^, Zn_2_P_2_O_7_:Sm^3+^, and ZnO-SO_4_:Sm^3+^ phosphor materials.

The FTIR spectra of ZnO:Sm^3+^, Zn_4_B_6_O_13_:Sm^3+^, Zn_2_P_2_O_7_:Sm^3+^, and ZnO-SO_4_:Sm^3+^ phosphors are depicted in Figure 7. The spectra are recorded in the wavenumber range from 400 to 4000 cm^−1^ measured at room temperature. The Zn-O bonds are revealed by the presence of a broad transmission band found between 450 and 650 cm^−1^ observed in the ZnO crystal structure materials [40,41]. The transmission bands around 3000–3700 cm^−1^ and around 1700 cm^−1^ observed in all samples are attributed to the O-H stretching modes of water moisture that settled on the sample surface during the fabrication process [14,42].

More transmission bands are observed for Zn_4_B_6_O_13_:Sm^3+^and Zn_2_P_2_O_7_:Sm^3+^ samples, which resulted in a different crystal structure from that of ZnO. For the Zn_2_P_2_O_7_:Sm^3+^ sample, characteristic transmission bands of symmetric and asymmetric PO_3_ functional groups are observed in the region around 1000–1200 cm^−1^ [43]. The symmetric modes of PO_3_ groups appear as a shoulder situated at 1078 cm^−1^ [44]. Similarly, the Zn_4_B_6_O_13_:Sm^3+^ FTIR spectrum shows three characteristic transmission bands at 600–700 cm^−1^, 800–950 cm^−1^, and 1200–1500 cm^−1^, which are attributed to B-O-B bending modes of BO_3_ and BO_4_ units, B-O stretching of the BO_4_ tetrahedral unit, and asymmetric stretching relaxations of the B-O bonds of the BO_3_ trigonal unit, respectively [45,46,47]. The transmission bands between 450 and 500 cm^−1^ are also attributed to BO_4_ units [42]. The FTIR analysis of ZnO-SO_4_:Sm^3+^ confirms the absence of characteristic sulfate ion vibrations (1100–1300 cm^−1^), indicating that sulfate species were not incorporated into the ZnO lattice. This observation is consistent with the XRD results [48], which show that ZnO remains the dominant crystalline phase. This suggests that during high-temperature combustion synthesis, the sulfate precursor decomposed and volatilized—likely as SO_3_ or SO_2_ gases. This finding corroborates that the detected 0.5 wt% sulfur in the EDS spectrum most likely originates from surface contamination or residual surface species.

### 2.4. UV–Visible and Band Gap Measurements

**Figure 8:**
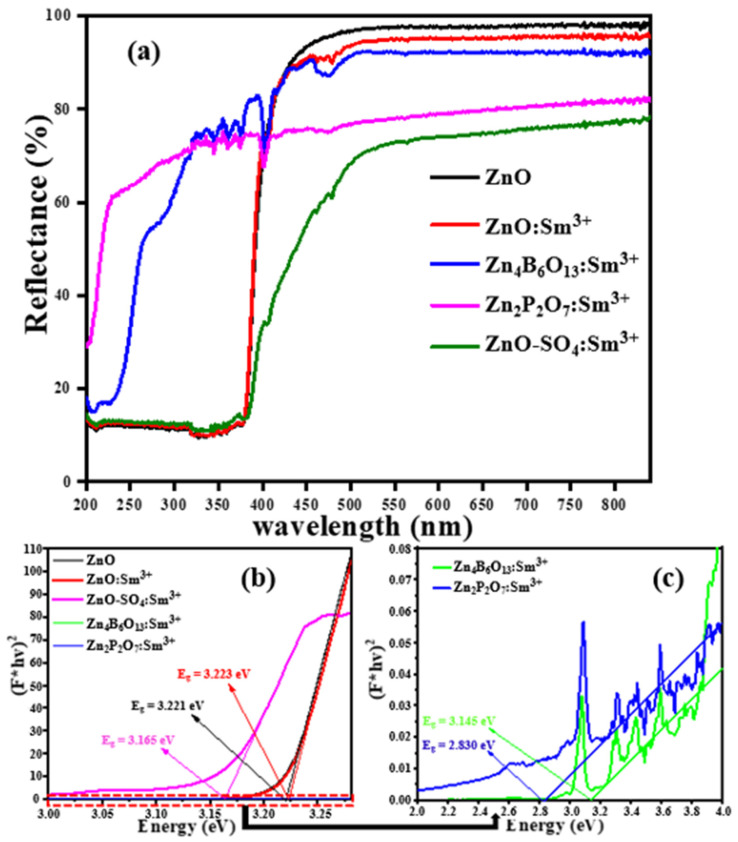
(**a**) UV-Vis spectra of ZnO, ZnO:Sm^3+^, Zn_4_B_6_O_13_:Sm^3+^, Zn_2_P_2_O_7_:Sm^3+^, and ZnO-SO_4_:Sm^3+^ phosphors material with 1mol% of Sm^3+^ ion, (**b**) optical gap of ZnO, ZnO:Sm^3+^, ZnO-SO_4_:Sm^3+^,Zn_4_B_6_O_13_:Sm^3+^, and Zn_2_P_2_O_7_:Sm^3+^ phosphors, and (**c**) enlarged figure showing the optical gap of the Zn_4_B_6_O_13_:Sm^3+^ and Zn_2_P_2_O_7_:Sm^3+^ phosphors.

The UV–visible reflectance spectra of undoped ZnO, ZnO:Sm^3+^, Zn_4_B_6_O_13_:Sm^3+^, Zn_2_P_2_O_7_:Sm^3+^, and ZnO-SO_4_:Sm^3+^ phosphor materials are presented in Figure 8a. The spectra are recorded in the spectral wavelength range from 200 to 800 nm. It is observed that all prepared materials show high and low reflectance intensities in the visible and UV regions, respectively. Similar reflectance behavior is observed for all materials with a wurtzite crystal structure, both in the visible and UV regions of the DSR spectra, and this is due to the similarity of their crystal structure. From these ZnO materials, the undoped ZnO has the highest reflectance intensity while the ZnO-SO_4_:Sm^3+^ has the lowest in the visible region. This is likely due to the sulfate volatilizing as SO_3_ during high-temperature synthesis, resulting in the crystal structure remaining intact but being influenced by residual sulfur contamination. The effect of Sm^3+^ doping is clearly observed in the DRS spectra of the ZnO:Sm^3+^ and ZnO-SO_4_:Sm^3+^ phosphor materials. The absorption band situated at 478 nm in these samples is due to the Sm^3+^ transitions. On the other hand, the other two substituted anionic groups, BO_3_^3−^ and PO_4_^2−^, which resulted in different crystal structures, also show different behavior in the DSR spectra as compared to the ZnO materials. The reflectance of these materials (Zn_4_B_6_O_13_:Sm^3+^and Zn_2_P_2_O_7_:Sm^3+^) lies deep in the UV region of the DSR spectra. The observed absorption bands for Sm^3+^-doped samples in the region 300 to 500 nm are attributed to the 4f^5^→ 4f^5^ intra-configurational transitions from the ground state (^6^H_5/2_) to various excited states ^3^H_7/2_ (345 nm), ^4^F_9/2_ (363 nm), ^4^D_5/2_ (376 nm), ^6^F_7/2_ (403 nm), ^4^I_13/2_ (365 nm), and ^4^I_11/2_ (478 nm) of the Sm^3+^ ion [16,49,50]. The presence of these bands shows that the atomic nature of Sm^3+^ is not affected much by the host material; only weak interactions are present between the host and the dopant ions [49]. This implies that there is an effective shielding of the forbidden 4f^5^ electrons by the filled 5s and 5p shells of Sm^3+^ ions.

The optical energies of ZnO, ZnO:Sm^3+^, Zn_4_B_6_O_13_:Sm^3+^, Zn_2_P_2_O_7_:Sm^3+^, and ZnO-SO_4_:Sm^3+^ phosphors were estimated from Kubelka–Munk (M-K) function theory by using data from Figure 8a [51]. The method adopted from the Tauc equation can be represented in the following form [51]:

(3) $${\left[F\left(R_{\infty }\right)hv\right]}^{2}=C(hv-E_{g})$$

Figure 8b,c show the plots of [$F(R_{\infty }).h\nu {]}^{2}$ as a function of photon energy hν for direct transitions. Extrapolations of the best linear parts of the curve to the x-intercept at zero absorption give the band gap energy values [52,53,54]. The estimated band gap values are presented in Table 3.

The band gaps for the pure ZnO, ZnO:Sm^3+^, Zn_4_B_6_O_13_:Sm^3+^, Zn_2_P_2_O_7_:Sm^3+^, and ZnO-SO_4_:Sm^3+^ phosphor materials appear to remain unchanged for the ZnO:Sm^3+^ sample, and this band gap becomes smaller for other samples when anionic groups are introduced, as tabulated in Table 3. Accordingly, by introducing various anionic groups, the band gap values are Zn_4_B_6_O_13_:Sm^3+^ (3.145 eV), Zn_2_P_2_O_7_:Sm^3+^ (2.830 eV), and ZnO-SO_4_:Sm^3+^ (3.165 eV) for the respective anions. The variation profile of band gap values clearly indicates that Zn_4_B_6_O_13_:Sm^3+^, Zn_2_P_2_O_7_:Sm^3+^, and ZnO-SO_4_:Sm^3+^ show lower band gap values compared to ZnO and ZnO:Sm^3+^ materials, with the Zn_2_P_2_O_7_:Sm^3+^ material having the smallest band gap value. The smaller decrease in the band gap of the ZnO-SO_4_:Sm^3+^ sample, which retained the hexagonal structure, is mainly due to the volatilization of the sulfate during synthesis, as observed from the DSR spectra. Numerical values are presented with estimated uncertainties derived from the instrumental resolution and data fitting precision of the optical band gap values, which carry a ±0.01–0.02 eV experimental error based on Tauc plot fitting.

### 2.5. XPS Analysis

**Figure 9:**
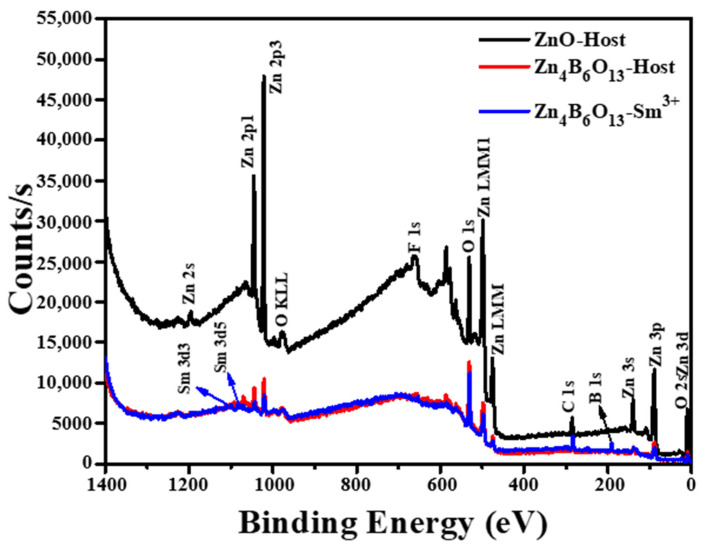
XPS survey of ZnO, Zn_4_B_6_O_13_, and Zn_4_B_6_O_13_:Sm^3+^ phosphors.

**Figure 10:**
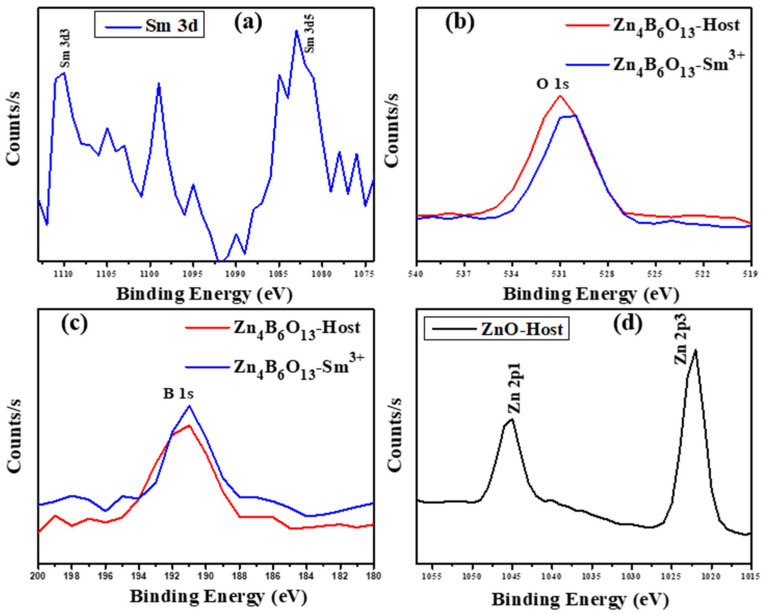
High-resolution XPS spectra of (**a**) Sm 3d, (**b**) O 1s, (**c**) B 1s, and (**d**) Zn 2p.

The XPS of ZnO, Zn_4_B_6_O_13_, and Zn_4_B_6_O_13_:Sm^3+^ were performed in order to assess the elemental states of the present elements in these materials. Figure 9 presents the XPS survey of ZnO, Zn_4_B_6_O_13_, and Zn_4_B_6_O_13_:Sm^3+^ phosphors recorded from a binding energy of 1400 to 0 eV. The composition of our samples is confirmed in Figure 9, as we can observe that the ZnO host sample contains core-level peaks from Zn 2p and O 1s, Zn_4_B_6_O_13_ shows core-level peaks from Zn 2p, O1s, and B 1s only, and Zn_4_B_6_O_13_:Sm^3+^ contains peaks from Zn 2p, O1s, B 1s, and Sm 3d [55,56]. This is in good correspondence with our EDS findings. Figure 10 shows high-resolution XPS spectra of Sm 3d, O 1s, B 1s, and Zn 2p bands from the prepared phosphors. From Figure 10a we observe two well-separated peaks of Sm 3d located at 1110.27 and 1082.95 eV, corresponding to Sm 3d_3/2_ and Sm 3d_5/2,_ respectively [57]. The binding energy difference of these two peaks is 27.32 eV, which confirms the +3-oxidation state of the Sm ion in the Zn_4_B_6_O_13_:Sm^3+^ sample [51,53,58]. The high-resolution spectra of O 1s and B 1s are represented in Figure 10b,c, respectively, for Zn_4_B_6_O_13_ and Sm-activated Zn_4_B_6_O_13_ samples. The binding energy curve of B 1s located at 191.02 eV is attributed to photoelectrons originating from B atoms bounded to oxygen (B-O) in the Zn_4_B_6_O_13_ matrix [56]. The Zn 2p XPS spectra, Figure 10d, reveal the Zn 2p_1/2_ and Zn 2p_3/2_ peaks located at a binding energy of 1045.01 eV and 1022.14 eV, respectively. These two peaks are characteristic of ZnO, and the binding energy difference of 22.87 eV also confirms the formation of ZnO material [55]. The peak located at a binding energy of 284 eV is attributed to carbon (C1s), which is due to the instrument; the C1s peak is normally used as the reference peak in determining the binding energies of other elements [59].

### 2.6. Photoluminescence Studies

**Figure 11:**
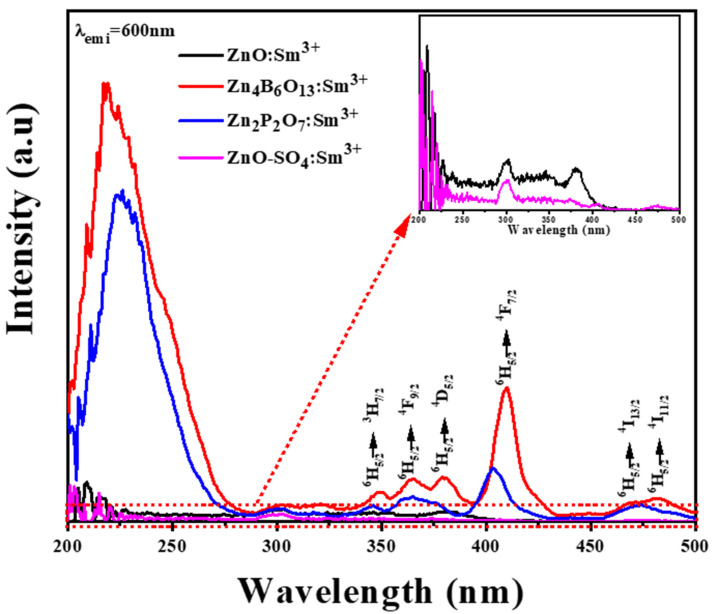
PL excitation spectra of ZnO:Sm^3+^, Zn_4_B_6_O_13_:Sm^3+^, Zn_2_P_2_O_7_:Sm^3+^, and ZnO-SO_4_:Sm^3+^ phosphor materials with 1 mol% of Sm^3+^ ion monitored under 600 nm wavelength.

The PL excitation spectra of ZnO:Sm^3+^, Zn_4_B_6_O_13_:Sm^3+^, Zn_2_P_2_O_7_:Sm^3+^, and ZnO-SO_4_:Sm^3+^ materials while monitoring at an emission wavelength of 600 nm are presented in Figure 11. The borate and phosphate materials show some intense excitation peaks as compared to other phosphor materials. The excitation band observed in the region between 200 and 275 nm can be associated with the charge transfer of Sm^3+^→ O^2−^ [14]. The intense excitation peaks are due to transitions from the ground state of Sm^3+^ (^6^H_5/2_) to various excited states such as ^3^H_7/2_, ^4^F_9/2_, ^4^D_5/2_, ^4^F_7/2_,^4^I_13/2_ and ^4^I_11/2_ located at 345 nm, 361 nm, 376 nm, 403 nm, 470 nm, and 486 nm wavelengths, respectively [60]. From these excitation peaks, the most intense peak observed at 403 nm is used as an excitation wavelength for the PL emission scan of these materials. The highest intensity for this transition is observed in the Zn_4_B_6_O_13_:Sm^3+^ sample.

**Figure 12:**
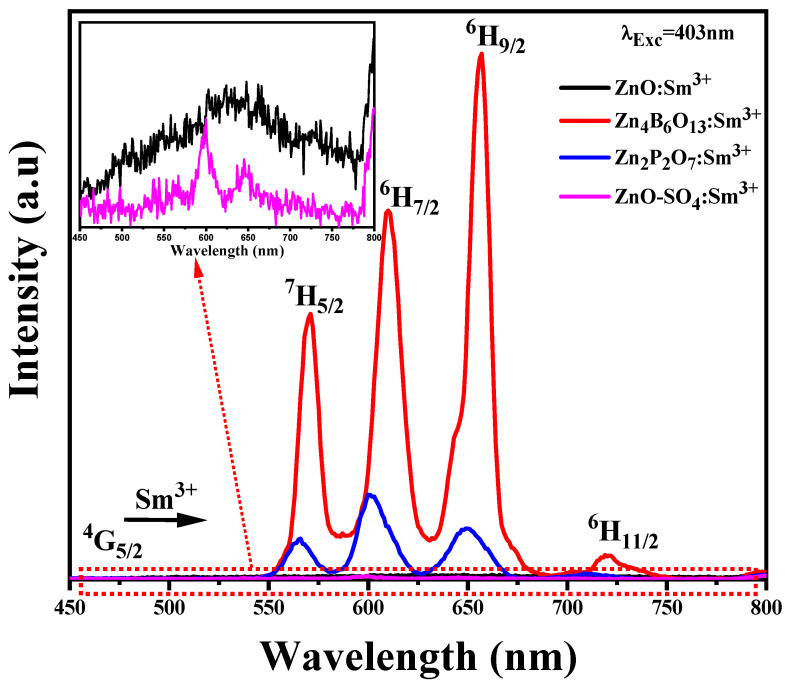
PL emission spectra of ZnO:Sm^3+^, Zn_4_B_6_O_13_:Sm^3+^, Zn_2_P_2_O_7_:Sm^3+^, and ZnO-SO_4_:Sm^3+^ phosphors material with 1 mol% of Sm^3+^ ion monitored under 403 nm excitation wavelength.

**Figure 13:**
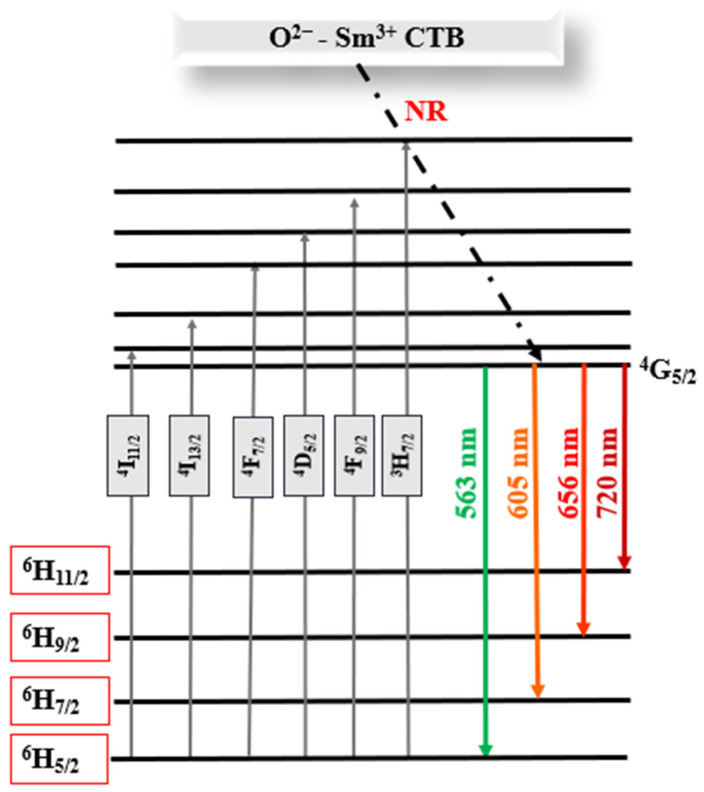
Partial energy level diagram of Sm^3+^ in Zn_4_B_6_O_13_:Sm^3+^ and Zn_2_P_2_O_7_:Sm^3+^ phosphors.

The PL emission scan for the prepared materials was carried out using an excitation wavelength of 403 nm (Sm^3+^ ion), and the spectra are displayed in Figure 12. In the wavelength region 400–800 nm, the characteristic Sm^3+^ emissions are only observed from the Zn_4_B_6_O_13_:Sm^3+^ and Zn_2_P_2_O_7_:Sm^3+^ samples, while the other materials show negligible emissions (see insert of Figure 12). The absence of characteristic Sm^3+^ emissions from ZnO:Sm^3+^ and ZnO-SO_4_:Sm^3+^ may be due to the host influences on the Sm^3+^ luminescence properties. The absence of characteristic Sm^3+^ emissions from ZnO:Sm^3+^ may be attributed to the strong influence of the ZnO host lattice defects on the luminescence properties of Sm^3+^ ions. In the ZnO-SO_4_:Sm^3+^, the low emission intensity could result from partial decomposition or volatilization of the sulfate species as SO_3_ during high-temperature synthesis, which alters the local chemical environment of Sm^3+^. Additionally, ZnO may induce non-radiative relaxation processes or inefficient energy transfer between the host and the Sm^3+^ activator, leading to luminescence quenching. Figure 13 shows a schematic representation of Sm^3+^ energy levels within the Zn_4_B_6_O_13_:Sm^3+^ and Zn_2_P_2_O_7_:Sm^3+^ phosphors. Firstly, when excited with 403 nm, the ^6^H_5/2_ level of Sm^3+^ transitions to a higher energy state, followed by non-radiative relaxations to the metastable level ^4^G_5/2_. Finally, the ^4^G_5/2_ level depopulation to the ground state results in different Sm^3+^ emissions [61]. The four characteristic Sm^3+^ emissions observed from Zn_4_B_6_O_13_:Sm^3+^ and Zn_2_P_2_O_7_:Sm^3+^ samples located at 563 nm, 605 nm, 656 nm, and 720 nm correspond to ^4^G_5/2_ → ^6^H_5/2_, ^4^G_5/2_ → ^6^H_7/2_, ^4^G_5/2_ → ^6^H_9/2_, and ^4^G_5/2_ → ^6^H_11/2_ transitions, respectively [14,60]. The highest intensity is observed from the Zn_4_B_6_O_13_:Sm^3+^ material. This high intensity may be attributed to the high crystallinity of the borate material. Of these Zn_4_B_6_O_13_:Sm^3+^ emissions, the most intense is from the ^4^G_5/2_ → ^6^H_9/2_ transition. The Zn_2_P_2_O_7_:Sm^3+^ sample, which has the second highest Sm^3+^ emission intensity, has the most intense peak located at 605, which is assigned to a ^4^G_5/2_ → ^6^H_7/2_ transition. From these Sm^3+^ emission bands, the emissions at 656 nm and 720 nm are attributed to the electric dipole (ED) contributions [14,49], while the other two emission bands at 605 nm and 563 nm are attributed to both electric and magnetic dipole (MD) contributions [14,49]. From the combinations of dipole contributions at these sites, the magnetic dipole contribution at ^6^H_5/2_ is most dominant, while the electric dipole contribution at ^6^H_7/2_ is most prominent [42].

The intensity ratio, which is the ratio of the electric dipole to the magnetic dipole, R = $\frac{ED}{MD}$, quantifies the symmetry position of the Sm^3+^ ion in the local surroundings of the host material. This ratio is a measure of distortion and is often called the asymmetric ratio (which is a measure of distortion) [5]. The measured intensity ratio for Zn_4_B_6_O_13_:Sm^3+^, R = I(G5/24→H9/27)I(G5/24→H5/27), is greater than 1, implying that Sm^3+^ ions occupies the asymmetric sites (absence of symmetry) in the crystal lattice at a fixed concentration of 1 mol% [5,49], leading to a higher ^4^G_5/2_ →^6^H_9/2_ emission for the ED contribution. In the case of the Zn_2_P_2_O_7_:Sm^3+^ phosphor material, R is slightly greater than 1 (R = 1.31). The high R value (1.95) for Zn_4_B_6_O_13_:Sm^3+^ compared to the Zn_2_P_2_O_7_:Sm^3+^ material implies that there is a greater asymmetrical property in the crystal field for the borate sample as compared to phosphate sample. The calculated R value of 1.95 for Zn_4_B_6_O_13_:Sm^3+^ is much closer to the calculated value of 2.05 by Zhang et al. on a different host material [20]. The effect of different host materials on Sm^3+^ ion emissions can be clearly seen from the PL peak shift. The Zn_4_B_6_O_13_ host material causes red shift, which implies that there is an energy transfer from the host material to the Sm^3+^ ions, and this can be confirmed by high-intensity emissions of the activator ion Sm^3+^ in the Zn_4_B_6_O_13_:Sm^3+^ sample.

### 2.7. CIE Chromaticity Coordinates and CCT Values

**Figure 14:**
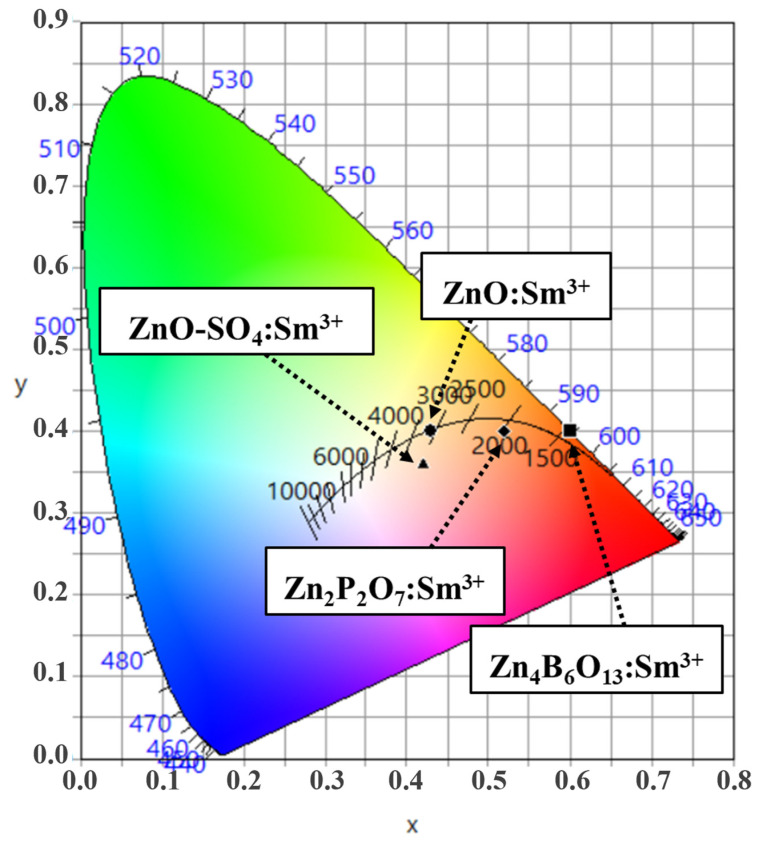
CIE chromaticity diagram of ZnO:Sm^3+^, Zn_4_B_6_O_13_:Sm^3+^, Zn_2_P_2_O_7_:Sm^3+^, and ZnO-SO_4_:Sm^3+^ phosphors.

The Commission International de I’Eclairage (CIE) chromaticity coordinates for the ZnO:Sm^3+^, Zn_4_B_6_O_13_:Sm^3+^, Zn_2_P_2_O_7_:Sm^3+^, and ZnO-SO_4_:Sm^3+^ phosphor materials are depicted in Figure 14. The CIE color chromaticity coordinates of the ZnO:Sm^3+^, Zn_4_B_6_O_13_:Sm^3+^, Zn_2_P_2_O_7_:Sm^3+^, and ZnO-SO_4_:Sm^3+^ phosphor materials are given as (0.430, 0.400), (0.600, 0.400), (0.520, 0.400), and (0.420, 0.360) coordinates, respectively. The ZnO-SO_4_:Sm^3+^ sample shows minimal changes compared to the ZnO:Sm^3+^, suggesting that the shift in CIE coordinates likely arises from the residual sulfur contamination during the synthesis process. Using the CIE coordinate values, the color purities (CPs) of the phosphor materials are evaluated using equation [54]:

(4) $$\mathrm{color}\;\mathrm{purity}\;(\mathrm{CP})\;=\;\frac{\sqrt{{\left(x-x_{i}\right)}^{2}+{\left(y-y_{i}\right)}^{2}}}{\sqrt{{\left(x_{d}-x_{i}\right)}^{2}+{\left(y_{d}-y_{i}\right)}^{2}}}\times \;100\%$$

where ($x_{d}$,$y_{d})$ are the CIE color coordinates of the dominant wavelength (edge of color gamut, extrapolating the line from the neutral white light coordinate point to intersect the CIE coordinate), (x, y) are the color chromaticity coordinates, and ($x_{i}$,$y_{i})$ are the standard color coordinates of neutral white light, taken as (0.333, 0.333) [5,61]. The evaluated color purity for each phosphor material is given in Table 4.

From the CIE diagram, it is observed that the Zn_4_B_6_O_13_:Sm^3+^ and Zn_2_P_2_O_7_:Sm^3+^ materials’ color emissions lie deep in the red-orange color. The color purity of these materials has been evaluated as 98% for Zn_4_B_6_O_13_:Sm^3+^ and 71% for Zn_2_P_2_O_7_:Sm^3+^, producing orange emissions. The strong orange emissions produced by Zn_4_B_6_O_13_:Sm^3+^ result from the Sm^3+^ concentration of 1 mol%; this suggests that by varying the concentration of the dopant, red emissions might be produced. Red emissions of the commercially available Y_2_O_3_:Eu^3+^ phosphor have the CIE coordinates of (0.622, 0.350), which are close to the results for Zn_4_B_6_O_13_:Sm^3+^ materials (0.600, 0.400) [62]. The Zn_4_B_6_O_13_:Sm^3+^ resulted in an orange emission of high quality. The ZnO:Sm^3+^ and ZnO-SO_4_:Sm^3+^ phosphor materials were evaluated to produce yellow emissions of 46% and 27% purity, respectively.

The additions of borate ions effectively resulted in orange luminescence of a high quality. In general, the chromaticity CIE coordinates (x, y) as well as chromaticity epicenter (x_e_, y_e_) = (0.3320, 0.1858) values were used to determine the correlated color temperature (CCT) values for the prepared phosphor materials by use of the McCamy empirical formula [63]:

CCT = 437n^3^ + 3601n^2^ + 6861n + 5517, where n = (x − 0.3320)/(y − 0.1858) (5)

The values obtained from this formula, together with their corresponding emission colors, are also shown in Table 4. The CCT values for the ZnO-doped materials were found to vary from 1713 K to 3090 K. These values correspond to typical temperatures of yellow to orange light emissions under near-UV excitation of light. The CCT value of the Zn_4_B_6_O_13_:Sm^3+^ material is regarded as a warm-orange-emitting phosphor candidate in W-LED applications.

### 2.8. Lifetime Decay Time Measurements

**Figure 15:**
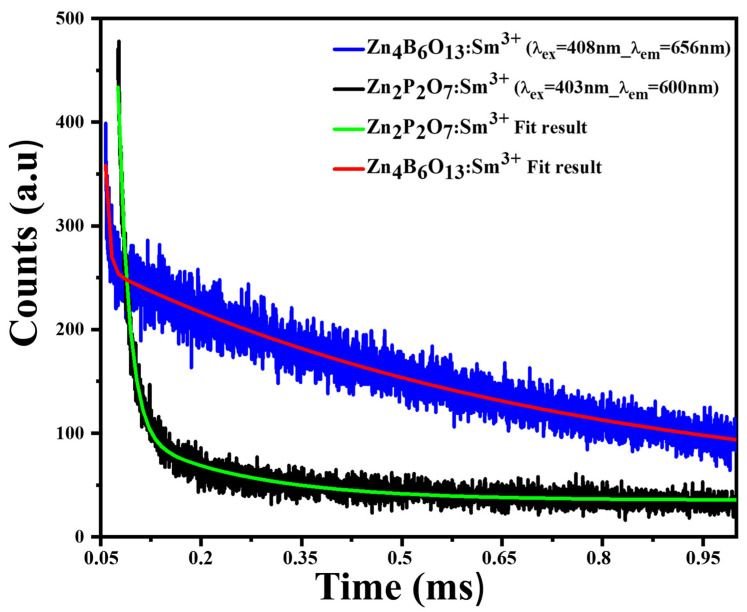
Lifetime decay curves for zinc metaborate and zinc pyrophosphate phosphor materials and their respective fit results.

Figure 15 shows the decay curves for Zn_4_B_6_O_13_:Sm^3+^ and Zn_2_P_2_O_7_:Sm^3+^ phosphor materials. The excitation wavelength used was 403 nm for both samples, and the emission wavelengths were 656 nm and 600 nm for Zn_4_B_6_O_13_:Sm^3+^ and Zn_2_P_2_O_7_:Sm^3+^, respectively. These were the most intense emissions; hence they were chosen. The two decay curves were well fitted by a bi-exponential curve of the form [5]

(6) $$I=I_{0}+A_{1}exp\left(\frac{-t}{\tau_{1}}\right)+A_{2}exp(\frac{-t}{\tau_{2}})$$

where $I_{0}$ and I are the luminescent intensities at t = 0 and t. Further, $\tau_{1}$ and $\tau_{2}$ are the fast and slow lifetimes, respectively. Fitting constants $A_{1}$ and $A_{2}$ are evaluated at time t, where $\tau_{1}$ = 0.6993244 ms, $\tau_{2}$ = 0.0051049 ms, $A_{1}$ = 217.58, and $A_{2}$ = 10.65 for Zn_4_B_6_O_13_:Sm^3+^ and $\tau_{1}$ = 0.1806959 ms, $\tau_{2}$ = 0.0161197 ms, $A_{1}$ = 620.81, and $A_{2}$ = 69.81 for Zn_2_P_2_O_7_:Sm^3+^. The equation used to find the average decay time of the ^4^G_5/2_ level for each phosphor material is given by the equation [5]

(7) $$\tau_{avg}=\frac{A_{1}\tau_{1}^{2}+A_{2}\tau_{2}^{2}}{A_{1}\tau_{1}+A_{2}\tau_{2}}$$

The average decay time for Zn_4_B_6_O_13_:Sm^3+^ and Zn_2_P_2_O_7_:Sm^3+^ phosphor materials was found to be 0.6991 ms and 0.1079 ms, respectively. The bi-exponential function indicates that the PL mechanism is linked to the trap states due to impurities or structural imperfections as dopants are introduced [64]. The significant difference in lifetimes may be due to the Zn_2_P_2_O_7_ favoring the non-radiative transitions due to the trap states’ recombination rather than the radiative transitions [64]. Furthermore, the overall decay time of Zn_4_B_6_O_13_ is in agreement with previous studies [65]. The shorter wavelength in the phosphate material arises from the high number of non-radiative transitions.

## 3. Materials and Methods

ZnO, ZnO:Sm^3+^, Zn_4_B_6_O_13_:Sm^3+^, Zn_2_P_2_O_7_:Sm^3+^ and ZnO-SO_4_:Sm^3+^ phosphors were prepared by urea-assisted combustion synthesis with a fixed concentration of Sm^3+^ at 1 mol%. ZnO-SO_4_:Sm^3+^ retained the wurtzite structure of ZnO when synthesized in the presence of ammonium sulfate, which acts as a combustion modifier rather than a dopant; the resulting product remains ZnO, as confirmed by XRD. The precursor materials for this method are in the form of nitrates (oxidizers), with the use of urea as a fuel (reducers). The high-purity raw materials were all sourced from SRL (RSA, Johannesburg, South Africa). In the preparation method, zinc nitrate hexahydrate Zn(NO_3_)_2_·(6H_2_O) (99%), urea (CH_4_N_2_O) (96%), and samarium (III) nitrate hexahydrate Sm(NO_3_)_3_·(6H_2_O) (99.9%) were weighed and added into different beakers at relevant masses to allow addition of anions. To the relevant masses, boric acid (H_3_BO_3_) (99.5%), di-ammonium hydrogen orthophosphate (N_2_H_9_PO_4_) (99%), and ammonium sulfate (H_8_N_2_SO_4_) (99.5%) were added to their respective beakers. De-ionized water (50 mL) was added to each beaker, and then the beakers were placed over a magnetic stirrer for 30 min at 500 rpm. The phosphate mixture showed a milky solution, and the other mixtures were clear. After stirring, the mixture was then placed in a pre-heated muffle furnace maintained at 600 °C for about 10 min, and a porous-like ash was produced. The ash was then ground with a mortar and pestle to produce greater homogeneity in the sample. The samples were then annealed at different temperatures for 5 h (undoped ZnO, ZnO:Sm^3+^, and ZnO-SO_4_:Sm^3+^ at 1000 °C, Zn_2_P_2_O_7_:Sm^3+^ at 600 °C, and Zn_4_B_6_O_13_:Sm^3+^ at 900 °C), after which they were ground again and then made ready for characterization.

The balanced chemical equations for the products are as follows:

Zn(NO_3_)_2_·(6H_2_O) + NH_2_CONH_2_ + (NH_4_)_2_HPO_4_ → Zn_2_P_2_O_7_ + gases (8)

Zn(NO_3_)_2_·(6H_2_O) + NH_2_CONH_2_ + (NH_4_)_2_SO_4_ → ZnO + SO_3_(g) + N_2_(g) + CO_2_(g) + H_2_O(g) (9)

Zn(NO_3_)_2_·(6H_2_O) + NH_2_CONH_2_ + H_3_BO_3_ → Zn_4_B_6_O_13_ + gases (10)

**Figure 16:**
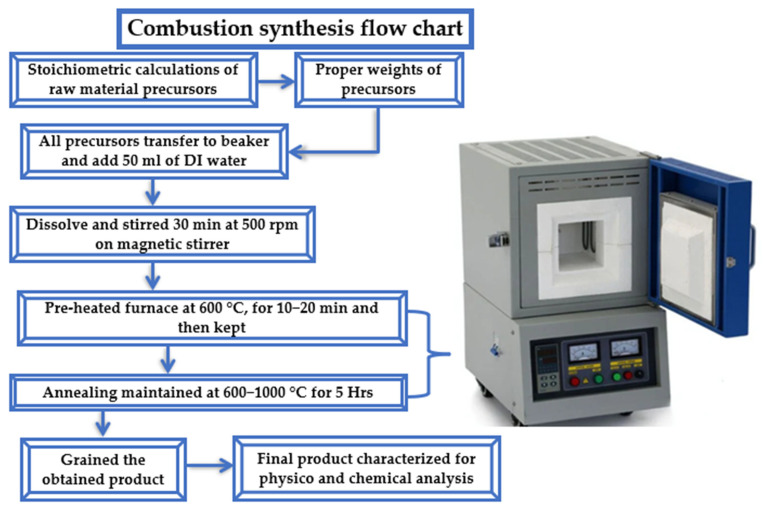
Flow diagram of combustion synthesis method.

It was observed in this synthesis process that the crystal structures of both phosphate and borate compounds changed, leading to different luminescent characteristics. Figure 16 shows a flow diagram of a typical combustion synthesis method.

## 4. Conclusions

This study successfully synthesized Sm^3+^-doped ZnO, Zn_4_B_6_O_13_:Sm^3+^, and Zn_2_P_2_O_7_:Sm^3+^ phosphors via combustion synthesis. While BO_3_^3−^ and PO_4_^3−^ formed new crystalline phases, the sulfate precursor decomposed, resulting in a ZnO lattice with minor surface sulfur traces (ZnO-SO_4_:Sm^3+^). Regarding XRD results, the formation of a hexagonal wurtzite crystal structure was observed for ZnO, ZnO:Sm^3+^, and ZnO-SO_4_:Sm^3+^ phosphor materials. However, incorporation of borate and phosphate ions resulted in the formation of cubic and monoclinic crystal structures, respectively. The cubic and monoclinic structures were observed for zinc metaborate (Zn_4_B_6_O_13_:Sm^3+^) and zinc pyrophosphate (Zn_2_P_2_O_7_:Sm^3+^), respectively. The PL emission scan monitored at an excitation wavelength of 403 nm revealed a characteristic emission of Sm^3+^ ions from the excited state ^4^G_5/2_ level to lower state ^6^H_J_ (J = 5/2, 7/2, 9/2, and 11/2) levels. A higher emission performance was observed for the Zn_4_B_6_O_13_:Sm^3+^ sample, followed by Zn_2_P_2_O_7_:Sm^3+^. An intense emission was observed for a peak at 654 nm. The lifetime measurement confirmed a higher decay time for Zn_4_B_6_O_13_:Sm^3+^ as compared to the Zn_2_P_2_O_7_:Sm^3+^ material. For color purity studies, the best color purity of 98% in the orange region was observed for Zn_4_B_6_O_13_:Sm^3+^ as the best color purity amongst all the prepared Sm^3+-^doped materials. This material can be regarded as a promising candidate for orange LED applications.

## Figures and Tables

**Table 1:** Estimated crystallite sizes of ZnO, ZnO:Sm^3+^, Zn_4_B_6_O_13_:Sm^3+^, Zn_2_P_2_O_7_:Sm^3+^, and ZnO-SO_4_:Sm^3+^ samples and their respective lattice parameters.

Sample	Crystallite Sizes (nm)	Lattice Parameters (Å)			Volume (Å^3^)
		a	b	c	V
ZnO	83.16 ± 0.85	3.2847 ± 0.001	3.2847 ± 0.001	5.2168 ± 0.002	48.74 ± 0.10
ZnO-Sm^3+^	83.13± 0.90	3.2600 ± 0.002	3.2600 ± 0.002	5.2347 ± 0.003	48.18 ± 0.12
Zn_4_B_6_O_13_:Sm^3+^	96.91 ± 0.95	7.4800 ± 0.003	7.4800 ± 0.003	7.4800 ± 0.003	418.51 ± 0.30
Zn_2_P_2_O_7_:Sm^3+^	59.75 ± 0.80	19.6170 ± 0.005	8.2780 ± 0.003	9.1060 ± 0.004	1454.19 ± 1.50
ZnO-SO_4_:Sm^3+^	73.33 ± 0.75	3.2540 ± 0.002	3.2540 ± 0.002	5.220 ± 0.002	47.87 ± 0.08

**Table 2:** Crystallographic data from Rietveld refinement of ZnO, ZnO:Sm^3+^, ZnO-SO_4_:Sm^3+^, Zn_4_B_6_O_13_:Sm^3+^, and Zn_2_P_2_O_7_:Sm^3+^ phosphors.

	ZnO	ZnO:Sm^3+^	Zn_4_B_6_O_13_:Sm^3+^	Zn_2_P_2_O_7_:Sm^3+^	ZnO-SO_4_:Sm^3+^
Crystal Structure	Hexagonal	Hexagonal	Cubic	Monoclinic	Hexagonal
Cell volume	47.49 ± 0.02 Å^3^	47.51 ± 0.02 Å^3^	417.73 ± 0.05 Å^3^	1442.1 ± 0.10 Å^3^	47.44 ± 0.02 Å^3^
Cell parameters	a = 3.243 Å	a = 3.24837 Å	a = 7.46348 Å	a = 20.056 Å	a = 3.25074 Å
	b = 3.243 Å c = 5.200 Å α = 90°, β = 90° γ = 120°	b = 3.24837 Å c = 5.20155 Å α = 90°, β = 90° γ = 120°	b = 7.48424 Å c = 7.47844 Å α = 89.9°, β = 90.1° γ = 89.9°	b = 8.241 Å c = 9.082 Å α = 90° β = 106.1° γ = 90°	b = 3.24470 Å c = 5.20453 Å α = 89.9°, β = 90.0° γ = 120.2°
R_p_	0.998	0.636	0.705	0.662	0.716
R_wp_	1.614	1.523	1.487	1.532	2.027
R_exp_	1.539	1.260	1.379	0.925	1.529
χ^2^	1.10	1.461	1.163	2.743	1.758
R-Bragg factor	11.516	10.952	16.488	12.716	16.064
R-Structure factor	7.181	7.944	14.001	10.131	14.764

**Table 3:** Band gap energies of ZnO, ZnO:Sm^3+^, Zn_4_B_6_O_13_:Sm^3+^, Zn_2_P_2_O_7_:Sm^3+^, and ZnO-SO_4_:Sm^3+^ materials estimated from the Kubelka–Munk plot.

Sample	Band Gap Energy (eV)
ZnO	3.221 ± 0.010
ZnO-Sm^3+^	3.223 ± 0.010
Zn_4_B_6_O_13_:Sm^3+^	3.145 ± 0.015
Zn_2_P_2_O_7_:Sm^3+^	2.830 ± 0.020
ZnO-SO_4_:Sm^3+^	3.165 ± 0.012

**Table 4:** CIE coordinates, color purity, and calculated correlated color temperature of ZnO:Sm^3+^, Zn_4_B_6_O_13_:Sm^3+^, Zn_2_P_2_O_7_:Sm^3+^, and ZnO-SO_4_:Sm^3+^ materials.

Sample	Chromaticity Coordinates				Color Purity (%)	CCT (K)	Color Range
	x	y	x_d_	y_d_			
ZnO:Sm^3+^	0.430	0.400	0.557	0.458	46	3090	Yellow
Zn_4_B_6_O_13_:Sm^3+^	0.600	0.400	0.606	0.402	98	1713	Orange
Zn_2_P_2_O_7_:Sm^3+^	0.520	0.400	0.606	0.402	71	1740	Orange
ZnO-SO_4_:Sm^3+^	0.420	0.360	0.672	0.347	27	2914	Yellow

## Data Availability

The raw data supporting the conclusions of this article will be made available by the authors on request.

## Abbreviations

The following abbreviations are used in this manuscript:

UV	Ultraviolet
NUV	Near Ultraviolet
LED	Light-Emitting Diode
PL	Photoluminescence
w-LEDs	White Light-Emitting Diodes
ASFAC	Ammonium Sulfate Fuel-Assisted Combustion
